# Sinusoidal obstruction syndrome (veno-occlusive disease) in a patient receiving bevacizumab for metastatic colorectal cancer: a case report

**DOI:** 10.1186/1752-1947-2-227

**Published:** 2008-07-11

**Authors:** Vijay Agarwal, Joseph Sgouros, Jacqueline Smithson, JPA Lodge, Abdul Razack, Anne Campbell, Anthony Maraveyas

**Affiliations:** 1 Academic Department of Oncology, Princess Royal Hospital, Hull, UK; 2 Gastroenterology Department, Hull Royal Infirmary, Hull, UK; 3 Hepatobiliary Unit, St James's University Hospital, Leeds, UK; 4 Radiology Department, Castle Hill Hospital, Cottingham, UK; 5 Department of Histopathology, Hull Royal Infirmary, Hull, UK; 6 Princess Royal Hospital, Hull and East Yorkshire Hospitals NHS Trust, Salthouse Road, Hull, HU8 9HE, UK

## Abstract

**Introduction:**

We present the case of a patient with colon cancer who, while receiving bevacizumab, developed sinusoidal obstruction syndrome (veno-occlusive disease) (SOSVOD). Certain antitumour agents such as 6-mercaptopurine and 6-thioguanine have also been reported to initiate hepatic SOSVOD in isolated cases. There have been no reports so far correlating bevacizumab with SOSVOD.

**Case presentation:**

A 77-year-old man was being treated with oxaliplatin and a modified de Gramont regimen of 5-fluorouracil for metastatic colon cancer. Bevacizumab (7.5 mg/kg) was added from the seventh cycle onwards. Protracted neutropenia and thrombocytopenia led to discontinuation of oxaliplatin after the ninth cycle. A computed tomography scan showed complete response and bevacizumab was continued for another 3 months, after which time the patient developed right hypochondrial pain, transudative ascites, splenomegaly and abnormal liver function tests. Upper gastrointestinal endoscopy showed oesophageal varices. Liver biopsy showed features considered to be consistent with SOSVOD. Bevacizumab was stopped and a policy of watchful waiting was adopted. He tolerated the acute damage to his liver and subsequently the ascites resolved and liver function tests normalised.

**Conclusion:**

We need to be aware that bevacizumab can cause sinusoidal obstruction syndrome (veno-occlusive disease) and that the occurrence of ascites should not be attributed to progressive disease without appropriate evaluation.

## Introduction

Severe sinusoidal obstruction syndrome (veno-occlusive disease) (SOSVOD) represents a life-threatening complication of dose-intensive chemotherapy. Conventional doses of certain antitumour agents such as 6-mercaptopurine and 6-thioguanine have been reported to initiate hepatic SOSVOD in isolated cases [1]. We present the first case of a patient with colon cancer who, while receiving bevacizumab, developed SOSVOD.

## Case presentation

**Figure 1:**
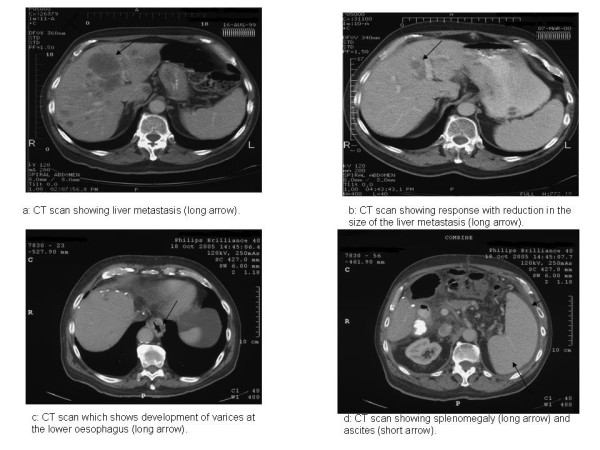
**CT scans**. **(a) **Liver metastasis (long arrow). **(b) **Response with reduction in the size of the liver metastasis (long arrow). **(c) **Development of varices at the lower oesophagus (long arrow). **(d) **Splenomegaly (long arrow) and ascites (short arrow).

A 77-year-old man presented with rectal bleeding in December 1998 due to carcinoma of the ascending colon and subsequently underwent right hemicolectomy, The tumour was staged as Dukes B. No adjuvant chemotherapy was given at that time. In August 1999 he developed disease recurrence in the liver (Figure 1a) and was treated with 12 cycles of oxaliplatin and a modified de Gramont regimen of 5-fluorouracil (OxMdg) with a good response (Figure 1b). In October 2000 he underwent left hepatic trisectionectomy, involving resection of hepatic segments 2, 3, 4, 5 and 8, and liver remnant metastasectomies of segments 1, 6 and 7, along with revision of the right hemicolectomy due to local recurrence. There was no evidence of chemotherapy-associated steatohepatitis in the hepatectomy specimen. His liver enzymes and synthetic function returned to normal after this.

He developed further extensive nodal recurrence both supra- and infra-diaphragmatically in November 2004 and was rechallenged with OxMdg. Bevacizumab at a dose of 7.5 mg/kg (675 mg) was added to the regimen from the seventh cycle onwards and was given every 4 weeks. Protracted neutropenia and thrombocytopenia led to discontinuation of oxaliplatin after the ninth cycle. His liver function tests at the time of discontinuing oxaliplatin were bilirubin 17 μmol/l, alkaline phosphatase 134 IU/l, alanine aminotransferase 87 IU/l and albumin 30 g/l. 5-fluorouracil was stopped after cycle 11 due to a computed tomography (CT) scan in July 2005 showing a complete response.

**Figure 2:**
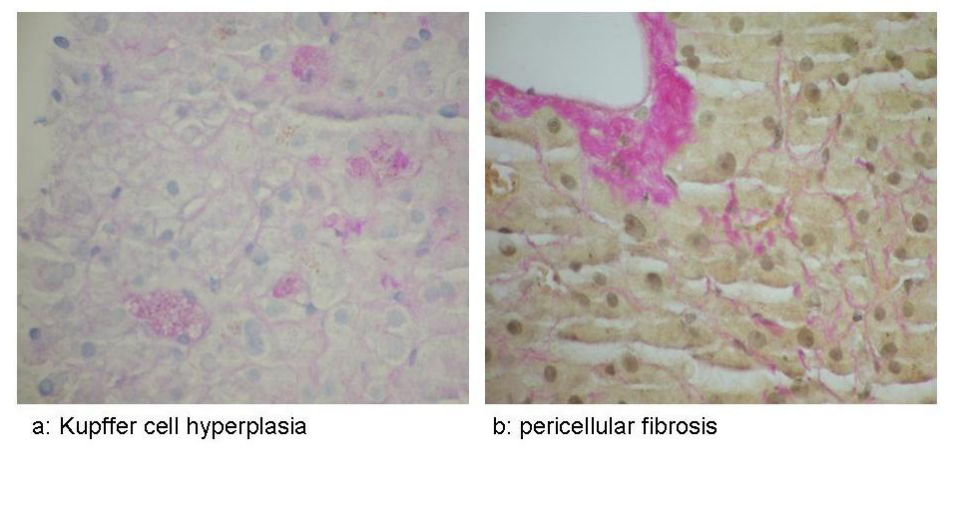
**PAS diastase and Van Gieson stains**. **(a) **PAS diastase stain showing Kupffer cell hyperplasia. **(b) **Van Gieson stain showing pericellular fibrosis adjacent to a terminal hepatic venule.

Maintenance bevacizumab (7.5 mg/kg every 4 weeks) was continued as a single agent. In September 2005 he developed right hypochondrial pain and liver function tests showed raised bilirubin 26 μmol/l, alkaline phosphatase 217 IU/l, alanine aminotransferase 99 IU/l and albumin 30 g/l along with persistent thrombocytopenia (<100 × 10^9^/l). He was not on any other medications which would significantly alter the liver function tests. CT scan showed oesophageal varices (Figure 1c), new ascites and splenomegaly (Figure 1d). The ascitic fluid was transudate with no evidence of malignant cells. Upper gastrointestinal endoscopy showed oesophageal varices, a Barrett's oesophagus and portal gastropathy. These findings were all consistent with an intrahepatic cause of portal hypertension. Liver biopsy showed Kupffer cell hyperplasia (Figure 2a) and pericellular fibrosis (Figure 2b) with a predominantly centrilobular distribution, but no definite occluded vessels were seen. There was no evidence of cirrhosis. These features were considered to be consistent with SOSVOD.

Subsequently, bevacizumab was stopped in October 2005 and a policy of watchful waiting was adopted. The patient recovered from the acute damage to his liver and subsequently the ascites resolved and liver function tests normalized. As of September 2007 the patient remains in complete remission but with persistent thrombocytopenia, splenomegaly and gastroesophageal junction varices.

## Discussion

There have been no reports so far correlating bevacizumab with SOSVOD. Bevacizumab blocks the circulating vascular endothelial growth factor (VEGF), which serves as an autocrine factor for the induction of neovascularization. Also it acts as a survival factor for tumour cells, protecting them from hypoxia, chemotherapy and radiotherapy. In normal tissues its action is stabilisation of mature cells. It has a beneficial effect in angiogenesis during wound healing [2].

Our patient had had a partial hepatectomy (trisectionectomy) in the past and had also received oxaliplatin-based chemotherapy, a drug recently found to cause hepatic sinusoidal dilatation in hepatectomy specimens. To date we have been unable to find any references on oxaliplatin associated with any clinical signs or symptoms of SOSVOD[3,4]. Blue liver syndrome as a consequence of VOD after the use of oxaliplatin has been reported by Bilchik et al. [5]. In our patient there was no evidence of blue liver syndrome in the hepatectomy specimen after the use of neoadjuvant oxaliplatin.

Potential explanations of the occurrence of the syndrome are possible blockage of the normal repair mechanisms induced by the VEGF, direct toxic action of the bevacizumab to the hepatic sinusoids or idiosyncratic and/or hypersensitivity reaction.

## Conclusion

We need to be aware that bevacizumab can cause sinusoidal obstruction syndrome (veno-occlusive disease) and that the occurrence of ascites should not be attributed to progressive disease without appropriate evaluation.

## Abbreviations

CT: Computed tomography; OxMdg: Oxaliplatin and modified de Gramont regimen of 5-fluorouracil; SOSVOD: Sinusoidal obstruction syndrome (veno-occlusive disease); VEGF: Vascular endothelial growth factor.

## Competing interests

The authors declare that they have no competing interests.

## Authors' contributions

All authors have been directly involved in the care of the patient. All authors have been involved in drafting or critically revising the manuscript for intellectual content and they have read and approved the final version submitted.

## Consent

Written informed consent was obtained from the patient for the publication of this case report and accompanying images. A copy of the written consent is available for review by the Editor-in-Chief of this journal.
